# Support needs of parents with preterm infants at resource-limited neonatal units in Limpopo province: A qualitative study

**DOI:** 10.4102/curationis.v46i1.2409

**Published:** 2023-10-26

**Authors:** Thendo Mahwasane, Khathutshelo G. Netshisaulu, Thivhulawi N. Malwela, Maria S. Maputle

**Affiliations:** 1 Department of Advanced Nursing Science, Faculty of Health Sciences, University of Venda, Thohoyandou, South Africa

**Keywords:** support needs, midwives, preterm infants, neonatal unit, discharge plan

## Abstract

**Background:**

Preterm birth is often unexpected and life-threatening for the baby and/or the mother. When admitted to the hospital, midwives need to provide informational, instrumental, psycho-cultural and emotional support to enhance post-discharge care.

**Objectives:**

This study aimed to explore and describe the support provided to parents of preterm infants in preparing for post-discharge care. The study was conducted in three district hospitals in the Mopani district, South Africa.

**Method:**

A qualitative approach wherein explorative, descriptive and contextual designs were used. A non-probability, convenience sampling was used to select 23 midwives who were working in the maternity unit for at least 2 years. Data were collected through in-depth individual semi-structured interviews until data saturation was reached. The data were analysed through Tesch’s open coding method. Trustworthiness was ensured through credibility, transferability and confirmability. Ethical principles adhered to were: informed consent, beneficence, right to self-determination, confidentiality and anonymity.

**Results:**

The findings revealed that parents need informational, instrumental direct supervision, and psycho-cultural and emotional support during preparation for discharge.

**Conclusion:**

Parents were unsure of their ability to care for the preterm infants after discharge and manage their own needs. The provision of informational, instrumental, psycho-cultural and emotional support needs would play a vital role in their ability to cope with their parental roles and the relationship with their infant.

**Contribution:**

The support provided to parents could build parental confidence and act as an integral part of neonatal follow-up programmes.

## Introduction

The birth and hospitalisation of a preterm infant are very distressing for parents (Mousavi et al. 2016) who undergo feelings of disappointment, fears regarding the child’s survival and altered parental experiences, including separation and reduced opportunities to interact with the infant; these are all difficult for parents. Trumello et al. (2018) concurred that preterm birth and the subsequent hospitalisation in the neonatal unit are considered early adverse experiences, which could affect the emotive states of the mothers, their mental representations, their perceptions of infants and their relationship in the early postpartum period including post-discharge. Depending on the degree of prematurity and the severity of the disease, the infant may have to be hospitalised for a long time in a neonatal unit (Kosowan et al. 2014). The prolonged stay of the preterm infants can be stressful for the mothers, and midwives should be there to support these mothers and ensure that they remain sane and are coping well with the situation. The World Health Organization (WHO) (UNICEF, WHO & World Bank 2014) indicated that in the United States (US) in 2007, the average length of stay for a preterm newborn (13 days) was nine times longer compared with a baby born at term (1.5 days). In South Africa, extremely low birth weight infants constitute 60% of the mortality rate related to preterm births (Ramokolo et al. 2019).

Parents are usually unfamiliar with the potentially complex problems their infants are facing, and they are unsure of their ability to care for the baby after discharge (Edéll-Gustafsson et al. 2015). In the context of this study, parents were not familiar with the neonatal unit staff; the language, routine and behaviours they encountered, contributed to an overwhelming feeling and hindered their participation in care. Parental stress is an important factor that affects a parent’s ability to care for their premature infant (Singer et al. 2010). Boykova (2016) concurred that parents with premature infants experience unique needs related to caring upon the initial discharge. These needs play a vital role in aspects such as their ability to cope with their parental roles and the relationship with their infant, their changing emotions and managing their own needs. Parents of premature infants require support in developing effective interactions with their infants. In support of the findings, Stefana and Lavelli (2017) indicated that the father’s capacity to participate actively in the care of the infant from the beginning is crucial in cases of premature birth. The well-being of the newborn, the development of mutual regulation, the establishment of a functional parent-infant affective relationship, and the parents’ confidence in their ability to care for their baby may all depend on how emotionally connected the parents are to the preterm infant being cared for in the neonatal intensive care unit (NICU). The need for informational and instrumental support is very important for mothers to cope with stresses related to prematurity and the neonatal unit environment. If knowledge about the support needs, stresses and challenges faced by mothers of preterm infants is to be effectively translated into practice, mothers need to be engaged differently, not just as informants about the problem of support but as co-carers (Sih, Bimerew & Modeste 2019).

Midwives and neonatal nurses experience ethical challenges when they interact with the parents of preterm infants during care provision; these may include emotional strain, protecting the vulnerable infant, ensuring continuity of care and miscommunication (Strandås & Fredriksen 2015). From the mothers, it was further noted that they lack the skill of feeding, practices of aseptic technique maintenance, some being afraid to hold their babies and practising their cultural beliefs within the neonatal unit that could cause complications, and become problematic and quired midwife attention. Mousavi et al. (2016) indicated the support necessary for midwives to provide to parents as instrumental, emotional, appraisal and informational-communicational. Appraisal support is defined as reinforcement for the parental role; mothers need to be close to the infant and have physical contact with him or her (Mousavi et al. 2016).

Midwives, as healthcare professionals, were directly providing care to preterm infants even though they encountered challenges related to the problems presented by the mothers. In the unit, midwives were ensuring the maintenance of thermoregulation, optimal positioning, airway clearance, stable haemodynamic status and adequate nutrition for growth and development (Joseph 2015). Midwives need to involve and upskill the mothers in preparation to continue with caring post discharge. The purpose of this study was to explore and describe the support provided to parents of preterm infants in preparation for post-discharge care.

## Research methods and design

### Study design

A qualitative research approach using exploratory, contextual and descriptive designs was conducted in neonatal units from February 2019 to July 2019 in selected hospitals in the Mopani District of the Limpopo province, South Africa. The district hospitals are situated in a rural area and were classified as resource-limited facilities. The hospitals were selected because of their high rate of neonatal births and mortality. The Limpopo province alone had 254 249 neonatal deaths with Mopani within the top three district with preterm births and mortality (Malwela & Maputle 2022) in 2006–2009. The socio-economic status of the mothers could have an impact on the increase in preterm birth and their survival, and appropriate discharge planning was imperative.

### Population and sample

The population comprised all midwives or accoucheurs who were working in neonatal section that provides care to preterm infants. Non-probability convenience sampling was used to select 23 midwives with at least 2 years’ experience of working in the unit. The reason for selecting midwives with 2 years or more of experience is that they have enough experience to share with the researcher.

### Ethical considerations

Ethical standards were ensured by obtaining the ethical clearance (ref: SHS/17/PDC/04/1403) from the University of Venda Ethics Committee and permission to conduct the study from the Limpopo Provincial Department of Health, the District Managers of the districts, the hospital chief executive officers (CEOs), and the participants. Participants were coded as participant A or any specific alphabet, and alphabet of the hospital. Midwives gave written, informed consent and were informed of their right to withdraw from the study without any penalty and that participation is voluntary. Ethical principles of fairness, privacy, confidentiality and anonymity as well as participants’ rights to voluntarily participate in the study were adhered to.

### Data collection

Data were collected through unstructured face-to-face interviews to gain a detailed picture of the support provided to mothers when the infant is admitted to the neonatal unit and how they are preparing the mother for discharge. Interviews were conducted in the duty room during their resting period. The questions asked were ‘*could you share with me about the support you are providing to mothers in preparation for their preterm infants*’ *post discharge care*?’ Probing questions were guided by the participants’ responses. The probing deepened the responses to questions and increased the richness of the data. Data were collected to the point at which no new information was obtained and data saturation was reached. The language used during data collection was English and lasted for about 30–45 min. Permission to record the interview was sought, and a voice recorder was used to capture the data.

### Data analysis

The narrative data from the in-depth individual interviews were analysed qualitatively using Tesch’s open coding method as postulated by Creswell and Creswell (2017) and De Vos et al. (2011). The recorded interviews were transcribed word by word and the non-verbal cues for example, silence and/or sigh, frowns and lean back; (De Vos et al. 2011) were also included. All transcripts were read to give meaning, and a list of similar topics was clustered. Data were grouped according to categories and sub-categories, initial 8 categories, and 3 subthemes were merged to form 1 theme. The field notes were also coded and categorised. The themes that emerged were supported by quotations from the interviews. A literature control was done to control the results of the study.

### Trustworthiness

The criteria for ensuring trustworthiness as outlined in Polit and Beck (2017) was adhered to. Credibility was ensured through prolonged engagement during data collection. The researcher met with the participant to establish rapport and to make an appointment. During the interviews, the researcher spent time with the participants listening and observing them as they were interviewed. The participants were interviewed to the point at which there was data saturation. A member check was also conducted to validate the truth and to confirm the findings. Voice recorder was used to ensure credibility. Transferability was ensured by thick descriptions of research methodology. The recorded interviews were transcribed verbatim and the nonverbal cues (e.g. silence/sigh, frowns and lean back) were included in brackets of the transcripts to ensure authenticity. Confirmability was ensured by documenting the detailed methodology and using an independent coder to confirm the generated codes. The author was questioned by supervisor to determine prejudices, and this was done to ensure bracketing, which was then used to direct and lessen biases in the ensuing.

## Presentation of findings

### Demographic data of participants

The total number of midwives who participated was 23 with at least 2 years of experience in providing care for preterm infants and their mothers. Of the 23 midwives who participated in the study, 95.7% were females and 4.3% were males. In terms of ethnic groups, 47.8% were Northern Sotho’s, 39.1% Tsongas, 8.7% Swati and 4.3% Venda. Regarding occupation, 4.3% were operational managers, and 95.7% were general midwives. None of them were trained for neonatal intensive care. The demographic profile of the participants did not have any difference in how midwives perceived the support needs of mothers when admitted to the unit. The findings revealed one theme as: support needs for mothers of preterm infants in preparation for post-discharge care, with three sub-themes: namely, the need for information, instrumental support needs through direct supervision, and psycho-cultural and emotional support needs (see Table 1).

**TABLE 1:** Presentation of a theme and sub-themes.

Main theme	Subthemes
Support needs for mothers of preterm infants in preparation for post discharge care	1.1 Informational support needs 1.2 Instrumental support needs through direct supervision 1.3 Pyscho-cultural and emotional support needs

### Theme 1: Support needs for mothers of preterm infants in preparation for post-discharge care

Parents and/or mothers of preterm infants need attention and information so that they understand the care provided to their infants and how to continue when discharged. The midwives need to support parents of preterm infants when providing the following to preterm infants: thermoregulation, optimal positioning, airway clearance, stable haemodynamic status and adequate nutrition for growth and development. This was supported by one of the participant who stated:

‘I have a day 37 preterm baby in the unit born at 0.85kg, current weight is 1.22kg … mother reported at the hospital in early labour and was given steroids … baby was born and admitted in neonatal unit. During the first days, the mother was too distant, couldn’t accept the baby, couldn’t change nappies, and always had to be reminded of the times to feed the baby. The mother was psychologically stressed. The mother was involved, and was provided with information on preterm care, did continuous counselling, and was referred to a psychologist several times. She was educated on the condition of the baby and what common clinical problems to look out for and told to report any change noticed in the baby. The mother now is able to independently provide care, participating in the management plan … and the baby has been off oxygen and feeds well with no problems.’ (Participant A, female, 24 years old)

Midwives also need to provide instrumental and emotional and/or psychological support. Under the theme, 3 sub-themes emerged: namely, informational, instrumental and psychological support needs, and each sub-theme is discussed next.

#### Sub-theme 1.1: Informational support needs

The provision of continuous and appropriate information to parents about the condition of their infant helps to ensure that the parents become more active partners and confident in taking care of their infants. The following quotations showed that parents of preterm infants need support:

Mothers need counselling from the day of admission. Sometimes a mother can see another baby with the same weight as her baby’s being transferred to the Kangaroo Mother Care [*KMC*] unit and hers still having drips, feeding tubes, etc., and start worrying and sometimes gets to a point of being psychotic. Therefore, the mother should be involved in every action to understand every management to be executed. Psychological distress in mothers affects breast milk production. Everyone nursing preterm infant should be patient and understand that care involves the mother.’ (Participant A, female, 22 years old)

Another participant said:

‘After some days of counselling mothers will reveal they were just scared of being around their babies because they are very small and not easy to hold, they feel like they’re hurting them when they hold them. This will confirm she can care to the infant post discharge.’ (Participant H, female, 21 years old)

It has been revealed that continuous counselling and involving the mothers in the care of their preterm infants can be one of the effective ways to successful management of preterm infants and readiness for post-discharge care. Midwives indicated the need for community members to be given information on the causes of preterm birth and the possible outcomes, so that they can support the mother. The following was cited to support this:

‘[*T*]he high rate of preterm birth is because of lack of knowledge of the community. The community is not aware of the causes and prevention of preterm labour. One woman said she was having an abortion … while some women attempt to abort a viable pregnancy at home and when they start bleeding, they are rushed to the hospital where they end up delivering live preterm babies.’ (Participant C, female, 28 years old)

If the community were to be been made aware of preterm birth, the complications and the care of an infant delivered prematurely, they may be able to provide the necessary support to a recently discharged mother having a preterm infant.

This is what one of the participants said related to information on post-discharge care:

‘Babies are discharged at 1.8 kg and should continue with skin-to-skin care at home and follow up at the hospital until the baby reaches the weight of 2 kg. Some mothers became reluctant when they got home and did not comply with this, and when they bring the baby for follow-up at two weeks you find that the baby has lost weight or is still on the same weight they were discharged at, this leads to re-admission.’ (Participant E, female, 25 years old)

Women should be provided with information on the importance of antenatal care. When pregnant women do not report the danger signs in pregnancy, it becomes a challenge as early detection of the complications of preterm birth, like premature rupture of membranes, may be missed. A participant responded in support:

‘Another challenge is with those mothers who present to the hospital in advanced labour when there is no time to intervene by stopping contractions with giving steroids to speed up lung maturity. Their babies will be born with severe respiratory distress and may also have recurrent apnoeic attacks.’ (Participant B, female, 27 years old)

Some of the mothers do not understand the importance of history taking and reporting of previous and current problems during antenatal care, which makes them think if they are going to the clinic and getting checked it is okay. They do not believe that the complications with their previous pregnancy can affect their current pregnancy. One of the participants said:

‘I have had a woman who delivered a preterm baby at 32 weeks gestation. On the antenatal cared nothing abnormal was indicated on the obstetric history, according to the notes recorded it was her first pregnancy. As I was nursing her baby and interviewing her, she revealed that she has had two miscarriages previously she didn’t report this to the midwives who saw her during antenatal care … she said she didn’t want those midwives to know as they were from the same village … It became obvious that her preterm delivery was due to her bad obstetric history … her cervix was incompetent and this could have been prevented if she had provided all the information during ANC [*antenatal care*].’ (Participant G, female, 32 years old)

Information on increasing access to care during pregnancy is an essential step towards addressing the growing problem of preterm birth (UNICEF, WHO & World Bank 2014).

#### Sub-theme 1.2: Instrumental support needs through direct supervision

Mothers need close monitoring as some do not understand how they should care for their infants. This is supported by the following quotation:

‘During feeding times, mothers need to be monitored closely to make sure they are not under [*and/or*] over-feeding their babies. It is possible to monitor all the mothers because there is a high shortage of staff. We show the mothers how to feed on the first days, and each morning feeds are being reviewed according to the age and weight of the baby, and each mother is supervised and monitored how much they feed until they master the skill. Some mothers will feel like they are not doing enough and end up adding more feeds to what has been prescribed and over-feed the baby, which causes vomiting and aspiration.’ (Participant B, female, 27 years old)

Dealing with young mother’s results in more problems than when it is someone matured. As a result, most preterm infants of teenage mothers are likely to complicate more because of the actions of their mothers. This was supported by one of the participants who stated:

‘[*T*]eenage mothers are still young and not matured. They are not able to take care of their babies, even when taught how to feed they don’t practice what they have been taught. They forget easily. Their babies are most likely to aspirate because they put them on the bed immediately after feeding … sometimes put the baby on fowlers position instead of lateral and since preterm babies are more likely to vomit, they get choked when they vomit and sometimes aspirate. When transferred to the KMC unit, they don’t practice skin-to-skin as they were taught … More attention is given to them.’ (Participant C, female, 24 years old)

Mothers need guidance, reinforcement and positive feedback when taking care of their infants.

#### Sub-theme 1.3: Pyscho-cultural and emotional support needs

Mothers believed it is a punishment to deliver a preterm infant. Midwives indicated how these mothers got these feelings:

‘Mothers who had experienced miscarriages or who have delivered premature babies are more affected than those who reached term pregnancy. It is very stressful to give birth to a preterm baby. The mother gets psychologically affected and ends up blaming herself, some mothers will end up saying that maybe they are being punished for the things they once did in the past. Sometimes the baby stopped breathing immediately after birth and need to be resuscitated for more than 30 minutes with no success, and this is very stressful.’ (Participant D, female, 26 years old)

One of the participants said this in support:

‘Mothers, especially first-time mothers who have just delivered a preterm baby become very stressed and traumatised because the baby will be very small with a very low birth weight … To mothers of babies who were referred from other hospitals often don’t have visitors because their homes will be far from the hospital. They get lonely and feel alone, they stress more when they see others being visited all the time.’ (Participant B, female, 22 years old)

Mothers become afraid of their babies and do not want to spend time around them because they are too tiny, and they view them as though they are not humans. They do not check on them as often as they should; they only come to see their babies when they are called by the midwives, and this results in a lack of bonding between the mother and the baby. This is supported by the following quotation:

‘Mothers of the preterm babies get scared of their babies because they are very small. They look at them as though they are not normal beings. They are afraid to hold them, change nappies, and bond with them, if not monitored closely they underfeed them just so they could leave the unit immediately after feeding or overfeed, and the baby will vomit and aspirate resulting in more serious conditions like choking and respiratory distress.’ (Participant F, female, 27 years old)

Even when the midwives encourage the mothers to stay with their infants, they still find a reason to leave the unit.

Cultural beliefs of the mothers of preterm babies interfered with the management of the babies and present a problem to the midwives. Participants verbalised this during interviews:

‘Another challenge is when the relatives try to sneak with herbal medications knowing that they are not allowed in the hospital … this is common to those who are in KMC [*Kangaroo Mother Care*] unit because midwives are not always in that unit but in the unit where there are very ill babies.’ (Participant H, female, 21 years old)

Another participant added:

‘[*T*]hey sometimes mix herbal medicine with breast milk so that nurses do not suspect anything … nurses will only realize after the baby starts changing condition and complicates. Some babies will even vomit and aspirate these herbal medicines. Some will have renal failure and distended abdomen and the mother will only report when she notices that the baby has not been passing urine for days.’ (Participant B, female, 22 years old)

Cultural health beliefs are still strongly practised, especially in developing countries on mothers and childcare. Psycho-cultural and emotional support are therefore important.

## Discussion

### Informational support needs

Parents who delivered premature infants experience unique needs related to caring for infants, if not met may have implications for continuing care when discharged (Boykova 2016). The knowledge deficit of mothers and community members about preterm care when discharged is problematic and leads to unpreparedness (Petty et al. 2019). The findings of the study confirm that parents often feel unprepared for the discharge to home, and thus they experience increased parental stress, worry and anxiety when they have to assume all the responsibilities of caring for their premature infant at home. Parents need information specific to premature infants and challenges related to infant feeding; infant behaviour and development; colic, crying and fussy periods; and how to recognise their infant’s changing health status. In support of the findings, one study indicated that both parents experience increased anxiety and stress during the first week of the infant’s discharge. Women were less anxious than men (Pace et al. 2016). Caring for premature infants leads to doubts, insecurity and fear among parents because they must become responsible for their care at home (Davis-Strauss, Johnson & Lubbe 2020). Similarly, Hemati et al. (2017) investigated the challenges of mothers after their premature infants were discharged home and found that parents with inadequate experiences or opportunities to care for their infants in the NICU had intensified fears and stress about caring for their infants’ post discharge. The midwives need to provide continuous and appropriate information and support to parents as this facilitates for parents to become active partners in taking care of their infants (Boykova 2016). This can be achieved by counselling and provision of information to mothers during pregnancy and after delivering the preterm infant as this could empower mothers to continue with care provision when discharged. Providing written information improves mothers’ understanding and recall (Muthusamy et al. 2012). Permitting the parents to be actively involved in neonatal units to care for their premature infants while under the supervision of the midwife would be an appropriate place to begin to ensure parents get the optimised support they need. Results also indicated that during their stay in the hospital, mothers need to be given continuous counselling by midwives and be referred to psychologists to improve their coping mechanisms with the situation of having a preterm baby. Sih et al. (2019) indicated that education of mothers regarding the care of their infants, the observation of mothers for psychological symptoms and referrals for psychotherapy and counselling could enhance the mother’s capabilities to cope with their challenges.

### Instrumental support needs through direct supervision

As perceived, parents need information and instrumental support to help them understand what happens on a day-to-day basis during the first few weeks and months following their discharge from hospital since they suddenly face unexpected challenges at home (Phillips-Pula et al. 2013). According to Hemati et al. (2017), mothers need more support on diapering, bathing, cord care, infant feeding, skin-to-skin care, recognition of the newborn’s cries and/or specialised care (e.g., medication administration, oxygen therapy and gastrostomy/colostomy care). While in hospital, they need close monitoring as some do not understand how they should care for preterm infants. Findings showed that it is vital for midwives to supervise mothers during feeding and when they practice Kangaroo Mother Care (KMC) to make sure that they are doing it correctly as taught. Midwives reported having experienced a challenge with the mothers who were in the KMC unit. Mothers were supposed to keep their babies in the skin-to-skin position to promote growth, prevent hypothermia, respiratory distress and to strengthen the bond between mothers and their preterm infants. Mothers did not comply with this; some did but they were not doing it correctly. Some mothers were found to be obstructing the babies’ nostrils with the breasts which can lead to more serious problems. The initial interaction between the mothers and their infants occurred under the supervision of the medical staff (Spinelli et al. 2016). Constant supervision by midwives was viewed to be critical. Skin-to-skin contact between the mother and their preterm infants not only encourages bonding but also allows the baby to be breastfeed at will and gives the energy to produce its heat (Hubbard & Gattman 2017). Kangaroo Mother Care is also used to prevent mortality and is a highly-yielding, low-tech, cost-effective intervention for addressing morbidity and mortality in preterm neonates (Ramokolo et al. 2019).

The daily infant care routines, combined with parental worries and stress, place high physical demands on the parents and often result in sleep deprivation, exhaustion and fatigue. The parents’ social lives are disrupted, which may lead to social isolation, difficulties in sharing feelings and misunderstandings from others (Phillips-Pula et al. 2013). Alderdice et al. (2018) found that adequate and professional healthcare services and access to additional support were not always available to parents after discharge. Premature infants often have ongoing health issues after being discharged to home, and often these problems result in rehospitalisation, neurodevelopmental complications and frequent follow-up medical appointments. This places an extra strain on the parents’ emotional well-being (Petty et al. 2019).

### Psycho-cultural and emotional support needs

Parents’ accounts of the experience of parenting a premature infant detail an emotional journey and a need for support during such a traumatic time; early in the experience, it appears parents need to adjust to the environment of the neonatal unit before they can focus on the needs of their infants (Wakely 2018). The midwives should consider parents’ need to adjust initially to the neonatal units’ environment before being able to focus on the needs of their infants. Findings indicated that some mothers believed it is a punishment to deliver a preterm infant. This was supported by Bravo and Noya (2014) who viewed labour complications as a punishment for a mother’s sins; thus, amid long and painful labour, they should focus on confessing their sins instead of seeking medical treatment. During the provision of care, mothers would add to the challenges of the midwives when they are psychologically traumatised and not responding well to counselling, and at times they would view their babies as though they are not human because of their birth weight. Mothers would perceive the infant as fragile and that they could be easily broken. Mothers’ trauma led to a variety of reactions, including sadness, fear, anger, grief, depression and helplessness (Purdy, Craig & Zeanah 2015). Study findings by Davis-Strauss et al. (2020) also revealed that when mothers’ interactions with their premature infants in NICU were hindered, it created feelings of confusion and detachment for the mothers. Midwives need to empathetically understand that even if the restriction of touch is medically best for the infant, this was distressing for the mother and they need to provide regular reassurance to the mother that their infant is developing well and may require help developing confidence with their parenting (Wakely 2018). González and Espitia (2014) found that on average, mothers needed 7 weeks to start feeling comfortable and secure in their care for their premature infants after the discharge to home. This implies that there were several challenges and changes in the mothers’ lifestyles, needs and expectations.

Findings from this study confirmed that parents with premature infants require optimal and ongoing support from midwives who are familiar with the unique needs of parents and premature infants. It was further noted that differences in cultural beliefs posed a great challenge during the care of preterm infants and their mothers. Mothers knew and understood that herbal medications were not allowed in the hospital premises, but they would still allow their relatives to bring them for babies. It was not possible for the midwives to always be around the mothers in the KMC unit because of staff shortage, which always made it difficult to monitor them closely. This allowed mothers to give their preterm infants herbal medications mixed with breast milk without the midwives’ knowledge. Preterm infants, because of their immature organs, face complications easily after being given herbal medications because all their systems are not fully matured including the renal, digestive and respiratory systems. Mothers only told the midwives about what they gave their preterm infants after they realised that their babies might face complications which may lead to their death. Midwives need to be sensitive to cultural practices among the population they serve. Some may be harmful, but may be beneficial to prevent mortality especially among the low-birth-weight babies (Sutan & Berkat 2014). Parents need more professional support and reassurance in their parenting and caregiving skills at home, as well as more information than what was provided in the hospital (Boykova 2016).

## Conclusion

Midwives identified the support needs of the mothers of preterm infants in preparation for post-discharge care as informational, instrumental support through direct supervision and psycho-cultural and emotional support. When opportunities for active participation are created, the provision of information could enhance the mother’s capabilities to cope with their challenges especially post discharge. However, within the limited-resource hospitals, this was not ideal because there were limited materials and human resources to provide constant support to the mothers.

### Recommendations

Midwives must adopt a holistic approach to care that acknowledges the uniqueness of each mother and supports them appropriately. A need for continued involvement and counselling of the mothers of preterm infants was emphasised, and it was shown to be the most vital in gaining the mother’s cooperation in the care of their infants. Cultural and religious variations may cause differences in the way experience of having a baby in the neonatal unit and the surrounding circumstances may be perceived and handled. Staff should be aware of this diversity so as not to view different responses by the parents as necessarily ‘abnormal’ or uncaring. The unit should establish the formal approach to discharge planning and to emphasise the psychological support as an integral part of neonatal follow-up programmes. The Department of Health needs to use evidence-based information to update its policies regarding the support needs of women nursing the admitted infants, neonates, child in the neonatal and/or children’s units of Limpopo province. Future researchers in the Limpopo province need to explore the gap regarding nursing mothers coping mechanism with the sick children admitted to hospitals.
